# Individual differences in auditory perception predict learning of non-adjacent tone sequences in 3-year-olds

**DOI:** 10.3389/fnhum.2024.1358380

**Published:** 2024-04-04

**Authors:** Jutta L. Mueller, Ivonne Weyers, Angela D. Friederici, Claudia Männel

**Affiliations:** ^1^Department of Linguistics, University of Vienna, Vienna, Austria; ^2^Vienna Cognitive Science Research HUB, Vienna, Austria; ^3^Department of Neuropsychology, Max Planck Institute for Human Cognitive and Brain Sciences, Leipzig, Germany; ^4^Department of Audiology and Phoniatrics, Charité – Universitätsmedizin Berlin, Berlin, Germany

**Keywords:** event-related potentials, sequence learning, non-adjacent dependencies, artificial grammar learning (AGL), auditory processing, infant development

## Abstract

Auditory processing of speech and non-speech stimuli oftentimes involves the analysis and acquisition of non-adjacent sound patterns. Previous studies using speech material have demonstrated (i) children’s early emerging ability to extract non-adjacent dependencies (NADs) and (ii) a relation between basic auditory perception and this ability. Yet, it is currently unclear whether children show similar sensitivities and similar perceptual influences for NADs in the non-linguistic domain. We conducted an event-related potential study with 3-year-old children using a sine-tone-based oddball task, which simultaneously tested for NAD learning and auditory perception by means of varying sound intensity. Standard stimuli were *A* × *B* sine-tone sequences, in which specific *A* elements predicted specific *B* elements after variable × elements. NAD deviants violated the dependency between *A* and *B* and intensity deviants were reduced in amplitude. Both elicited similar frontally distributed positivities, suggesting successful deviant detection. Crucially, there was a predictive relationship between the amplitude of the sound intensity discrimination effect and the amplitude of the NAD learning effect. These results are taken as evidence that NAD learning in the non-linguistic domain is functional in 3-year-olds and that basic auditory processes are related to the learning of higher-order auditory regularities also outside the linguistic domain.

## Introduction

An important part of auditory cognition is the ability to build relations between sounds that do not occur in direct sequence, but are separated by other intervening sounds. The extraction and processing of such non-adjacent dependencies (NADs) has been suggested to operate both in the auditory-perceptual and linguistic domain (Peña et al., 2002; Gebhart et al., 2009; Bendixen et al., 2012; Mueller et al., 2012; Wilson et al., 2018, for review; Winkler et al., 2018). A specific case from the auditory-perceptual domain that often includes processing of NADs is the phenomenon that listeners need to split rapid sequences of variable sounds into different perceptual streams, depending on various physical characteristics of the stimuli (Bregman and Campbell, 1971). In everyday life, auditory streams which indicate objects or specific events are often interrupted by other sounds. One might hear, for example, songs of different birds and in order to identify the sounds of one specific species, the listener has to extract sequential patterns across intervening sounds from other species. The linguistic domain, which manifests mainly in speech in everyday life, involves the ability to use NADs in similar ways. NADs are basic building blocks of human language and occur in many grammatical constructions, for example, “The **cat** that is strolling on the roofs **is** very old.” Such dependencies can occur between syntactic categories (in the example, noun and verb) or between specific morphemes (in the example, the third person and singular – s).

The learning mechanisms that have been suggested to underlie the human ability to extract sequential patterns from auditory input are statistical learning of transitional probabilities and rule-learning supported by non-distributional, perceptional cues (Endress and Bonatti, 2016). Evidence for both accounts will be reviewed to motivate the present investigation, but since this study is not designed to evaluate these two accounts, the general terms “artificial grammar learning” and “NAD learning” will be used in the following without any commitment to a specific mechanism supporting this learning process. Human sequence learning abilities have been shown to be domain-general on the one hand, and tuned to specific input signals, for instance language, on the other hand. Domain-generality is attested by many experiments demonstrating that statistical learning works in the auditory, visual, and even tactile domain (Saffran et al., 1996; Kirkham et al., 2002; Conway and Christiansen, 2005). This shows that in principle, learning takes place across different modalities and materials. Yet, many experiments also attest a modulation of artificial grammar learning processes dependent on the input modality and the specific type of (linguistic) materials (Frost et al., 2015; Milne et al., 2017; Rabagliati et al., 2019; van der Kant et al., 2020; Weyers and Mueller, 2022; Weyers et al., 2022). On top of modality and stimulus differences, individual differences have been found, suggesting, for instance, that statistical learning may be considered a well-defined cognitive ability (Siegelman and Frost, 2015). Other artificial grammar learning studies suggest that interindividual differences in basic auditory abilities may have an impact on performance in auditory artificial grammar learning tasks (Mueller et al., 2012, 2019; Studer-Eichenberger et al., 2016; Vasuki et al., 2017). In light of the mentioned potential domain-specificity of sequence learning abilities in the language domain and the perceptually based individual variation, we here aim to investigate whether the learning of NADs in early childhood, as previously investigated using speech materials (Mueller et al., 2012, 2019), shows similar characteristics in the non-linguistic auditory domain and, crucially, whether learning success shows similar relations to auditory perception as it does for speech materials.

When and how infants start to extract NADs is indicative of how infants approach the problem of acquiring structural knowledge in language. One method to assess whether and how NADs can be extracted by learners of different age groups is the measurement of electrophysiological (EEG) or hemodynamic (functional near-infrared spectroscopy; fNIRS) responses during natural language processing or artificial grammar learning experiments. These studies show that when exposed to sequences of speech sounds, infants are able to remember repetitions of vowels and transitions between adjacent syllables in the first month of life (Teinonen et al., 2009; Benavides-Varela et al., 2011, 2012). Other studies have, moreover, shown that starting at the age of 3 months, infants are also sensitive to more complex NADs between syllables (Friederici et al., 2011; Mueller et al., 2012; Friedrich et al., 2022). Behavioral indicators of successful NAD learning from artificial grammars have been reported from the first birthday (Gòmez and Maye, 2005; Lany and Gòmez, 2008; Culbertson et al., 2016), including the processing of word-internal phonological NAD relations (Gonzalez-Gomez and Nazzi, 2012, 2016). The detection of such structures in infants’ native language has been attested behaviorally from around the age of 18 months (Santelmann and Jusczyk, 1998; Hoehle et al., 2006).

When it comes to the question of whether similar learning processes are also present in the non-linguistic auditory domain, behavioral studies provide in principle affirmative evidence, although with important stimulus-dependent variation. Saffran et al. (1999), for instance, showed that 8-month-olds learn transitional probabilities between tones equally easily as those between syllables. Yet, Marcus et al. (2007) observed that 7-month-old infants could learn a repetition rule instantiated with tones and animal sounds only when the rule was presented after sequences of speech sounds. At the neurophysiological level, event-related potential (ERP) studies within the first half year of life indicate that the ability to extract adjacent as well as non-adjacent regularities is present both for speech (Teinonen et al., 2009; Friederici et al., 2011; Mueller et al., 2012) and non-speech auditory stimuli (Kudo et al., 2011; Winkler et al., 2018). Yet, there is evidence that the sensitivity to specific types of stimuli presented during such learning tasks is subject to developmental changes across the first years of life. Several studies have shown that very young infants in the first half year of life learn dependencies that are not, or not so easily, learned at later ages (Dawson and Gerken, 2009; Mueller et al., 2012, 2019). A study using fNIRS showed different trajectories for learning NADs from linguistic and non-linguistic sequences (van der Kant et al., 2020). While NADs were learned at age 2, but not at age 3 when presented in the form of artificial linguistic stimuli, NADs were learned successfully at 3 years of age, but not yet at 2 years of age, when presented in the form of non-linguistic tone sequences (van der Kant et al., 2020). This apparent decline in the ability to extract linguistic NADs around age 3 was further confirmed in an ERP study using the same linguistic stimulus material (Paul et al., 2021). On the basis of these findings, one may speculate that the learning of NADs in the non-linguistic domain may be present across development and not decline as it does in the speech domain. Yet, the scarcity of developmental data does not allow for firm conclusions about the developmental trajectory of NAD learning in linguistic versus non-linguistic auditory domains.

While it may be the case that the learning of statistical regularities such as NADs is specifically honed to the speech signal during early development, there is ample evidence that basic auditory processes impact on the processing and learning of grammatical dependencies, as evidenced in studies both with impaired and unimpaired populations (Halliday and Bishop, 2006; Bishop, 2007; Arciuli and Simpson, 2012; Mueller et al., 2012; Kidd and Arciuli, 2016). More specifically, children with language impairments (e.g., developmental language disorders or developmental dyslexia) often display deficits also with respect to the processing of basic auditory parameters, as for example duration processing (Corriveau et al., 2007) or frequency discrimination (Hill et al., 2005; Halliday and Bishop, 2006). For example, a longitudinal study with children with and without family history of language impairment showed that early differences (from 6 to 48 months) in the maturation of auditory evoked potentials in response to non-linguistic auditory stimuli predicted language abilities at 3 and 4 years of age (Choudhury and Benasich, 2011). What is more, children and adults with developmental language disorders show difficulties in the domain of auditory statistical learning in particular (Evans et al., 2009; Kahta and Schiff, 2019). At the other end of the continuum, it has been shown that musically trained children, who possess particularly rich auditory experiences, outperform untrained children in some language and statistical learning tasks (Kraus and Chandrasekaran, 2010; Francois and Schön, 2011; Vasuki et al., 2017). Taken together, the reviewed evidence suggests that both speech and non-speech sequence learning are linked to the quality of auditory processing.

While many studies have examined how the processing of specific acoustic parameters, for example, frequency (Halliday and Bishop, 2006; Mueller et al., 2012; Halliday et al., 2014) or duration (Weber et al., 2005) are linked to language processing, it remains unclear whether the relation between the quality of auditory processing and higher order cognitive processing holds for non-linguistic sequence learning in a comparable way. On the one hand, it is possible that specific auditory parameters are predictive of language abilities due to their importance for coding linguistic information (for example, frequency information determining vowel quality, or duration parameters contributing to word stress). For example, in the study of Mueller et al. (2012), pitch perception was found to be related to the learning of NADs between syllables. As frequency is not merely an auditory parameter, but also involved in how different vowels are coded, the link between auditory perception and NAD learning in this study could be explained because both processes are based on frequency discrimination. In this line of argumentation, auditory parameters that do not code distinctive features important for dependency learning should thus not be related to sequence learning. On the other hand, it is conceivable that rather domain-general mechanisms, for instance bottom-up auditory attention (Kaya and Elhilali, 2014; Addleman and Jiang, 2019), form the common basis for the processing of both speech and non-speech structured sequential inputs. Such domain-general attentional processes might be necessary for both rather low-level auditory processing and higher-level speech-based and non-speech-based processing of auditory sequences. Individual differences with respect to these attentional processes may affect neural correlates of processing in both domains. Under such a domain-general account, a variety of auditory parameters that render stimuli salient and attract listeners’ attention should be linked to sequence learning not only in the speech domain, but also in the non-speech domain. An example for such a parameter would be a change in intensity in sequences in which intensity is not relevant for the detection of a sequential regularity that has to be learned.

In the present study, we constructed NADs between non-linguistic pure tones and presented them in an oddball design, similarly to how Mueller et al. (2012, 2019) tested for syllable-based NAD learning. Using electroencephalography, we examined 3-year-old children’s ability to differentiate frequently presented standard exemplars of these tone NADs from infrequent deviant patterns. Three-year-olds were deemed a particularly relevant research population, because a beginning decline in NAD learning for speech stimuli had previously been reported for this age group (Mueller et al., 2019; van der Kant et al., 2020; Paul et al., 2021). As we specifically aimed to explore a possible impact of auditory processing abilities on non-speech NAD learning, we presented auditory deviants, which were reduced in intensity, in addition to NAD deviants within the same continuous stimulus stream. We hypothesized (i) that 3-year-olds would still be able to learn non-linguistic NADs, given a possible language- or speech-specificity of the previously established developmental decline in NAD learning ability (van der Kant et al., 2020). In the present experimental design, successful learning can be inferred from a reliable mismatch response (MMR) in the ERP with either positive or negative polarity. We further expected (ii) that successful NAD learning would be indexed by similar ERP patterns as reported for the speech domain, because a negative MMR for violated NADs compared to standard sequences was found in Mueller et al. (2019), in a design comparable to the present study; and (iii) that this ERP effect would be modulated by the individual ability to detect acoustic changes in the stimuli (cf. Mueller et al., 2012). In the present study, we chose to test intensity changes in order to test the above outlined possibility that domain-general, stimulus-driven, attentional mechanisms may be sufficient to explain a potential link between auditory processing and NAD learning. We expected this link to be indicated by a predictive relationship between the amplitude of the intensity-related MMR and the amplitude of the NAD-related MMR. Such a relationship should be specific to auditory change discrimination and not just reflect unspecific interindividual differences in auditory processing *per se*, which have been identified as an important source of variance across different perceptual and cognitive domains in ERP research. Specifically for auditory stimuli, it has been shown that amplitudes of the evoked signals vary considerably across individuals (Melnik et al., 2017). To control for an impact of such unspecific interindividual variation, we also tested for a relation between amplitude variations in response to standard stimuli alone and the NAD learning effects. Such a control will allow for more compelling evidence in case of a relationship between the auditory discrimination effect and the NAD learning effect.

## Materials and methods

### Participants

Our study was approved by the Ethics Committee of the University of Leipzig and conformed to the guidelines of the Declaration of Helsinki (World Medical Association, 2013). Before the experiment, parents and children were verbally informed about the test procedure and the accompanying caregiver gave written informed consent. All participants had normal hearing and no history of neurological disorders, were born between the 38th and 42nd week of gestation and were German monolinguals.

Of the 49 infants who participated in the study, data from 23 children (15 female) were excluded from analysis for the following reasons: children did not want to participate in the EEG measurement or wanted to stop it before the experiment ended (*n* = 7), data loss because of experimenter error (*n* = 1), children had been diagnosed with or were at risk for a developmental disorder (*n* = 2), high artifact rate (*n* = 13). The remaining 26 participants (7 female) had a mean age of 36.8 months (SD = 0.4).

### Experimental design and stimuli

**FIGURE 1:**
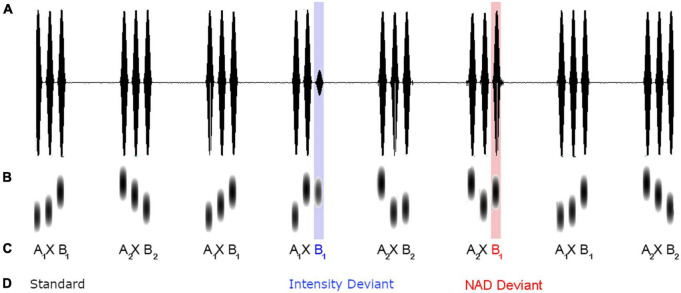
Schematic illustration of intensity **(A)**, frequency **(B)**, sequential structure (**C**, two types of NAD are indexed with the numbers 1 and 2 in the subscript) and condition of tone stimuli **(D)** embedded within an example of a presentation sequence.

The study made use of a modified oddball paradigm with triplets of sine tones. As illustrated in Figure 1, standard triplets conformed to an *A* × *B* grammar of item-specific NADs, in which the first tone *A* of a triplet reliably predicted the third tone *B*, while the intervening tone × varied. The individual tones covered a frequency range of 600–1,750 Hz in steps of 50 Hz and each tone had a duration of 100 ms. Two different *A* × *B* dependencies were used. The first type, *A*_1_ × *B*_1_, comprised an NAD between a 600 and a 1,450 Hz tone, the second type, *A*_2_ × *B*_2_, an NAD between a 1,750 and a 900 Hz tone. The middle × element was filled with the remaining set of 20 different tones (650, 700, 750, 800, 850, 950, 1,000, 1,050, 1,100, 1,150, 1,200, 1,250, 1,300, 1,350, 1,400, 1,500, 1,550, 1,600, 1,650, and 1,700 Hz) in a pseudorandom manner. Within each *A* × *B* triplet, tones were separated by 50 ms pauses, and between triplets there was a pause of 700 ms. A total of 80% of trials were standard stimuli, 10% were NAD deviants in which the NAD between *A* and *B* was violated (e.g., *A*_1_ was incorrectly paired with *B*_2_), and another 10% of trials were acoustic deviants, which were characterized by an intensity decrease of 25% (reduction from 82.27 to 62.83 dB).

Stimuli were presented according to a randomization scheme ensuring that each type of *A* × *B* triplet occurred with equal frequency in a given series of standards and that the same *A* × *B* triplet was not repeated more than three times in a row. Between two deviants, sequences consisting of two, four, six, or eight standard stimuli occurred. When standard sequences were shorter than four standards, different deviant types occurred before and after in order to provide enough exemplars to re-establish the correct *A* × *B* dependency. In total, 818 stimuli were presented, with 658 standard stimuli conforming to the *A* × *B* dependency and 80 exemplars of NAD deviants and intensity deviants each.

### Procedure

During stimulus presentation, children sat on their caregiver’s lap in a sound-attenuated testing booth. Caregivers wore sound-attenuating earplugs. Stimuli were played via two loudspeakers at the same comfortable sound level across participants. During the experiment, children were presented with a silent animation movie in order to prevent excessive movements of body and head (for a similar procedure, see Männel and Friederici, 2011; Männel et al., 2013). The whole experiment took approximately 14 min.

### EEG recording and analysis

The continuous electroencephalogram (EEG) was recorded from 23 Ag/AgCl monopolar electrodes fixed in an elastic cap placed on the child’s head (EASYCAP GmbH, Germany) at the following standard positions corresponding to the international 10–20 system (Jasper, 1958): F7, F3, Fz, F4, F8, FC3, FC4, T7, C3, Cz, C4, T8, CP5, CP6, P7, P3, Pz, P4, P8, O1, O2, M1, and M2. Signal recorded from the electrode positions F9 and F10 served to calculate the horizontal electrooculogram. The vertical electrooculogram was recorded from the electrode FP2 and an additional electrode placed below the right eye. FP1 served as the ground electrode, and Cz as online reference. Electrode impedances were largely kept below 10 kΩ (at least below 20 kΩ). The EEG signal was amplified with a gain of 20 using a PORT-32/MREFA amplifier (Twente Medical Systems International B.V.) with an input impedance of 1,012 Ω, and digitized online at 500 Hz (AD converter with 22 bit, digital filter from DC to 125 Hz). The following pre-processing steps were performed using MATLAB (version R2018b; The MathWorks Inc., 2018) and the EEGLAB open source toolbox (version 14.1.1b; Delorme and Makeig, 2004). Offline, the EEG was re-referenced to the average of both mastoids (M1 and M2). The continuous EEG was epochized from −200 to 800 ms relative to the onset of the final tone in each triplet and band-pass filtered between 0.5 and 30 Hz (digital windowed sinc FIR-filter, −6 dB half-amplitude cut-off, cut-off frequencies of 0.6 and 29.99 Hz). For the correction of eye-movements, the data were band-pass filtered between 1 and 30 Hz (−6 dB, cut-off frequencies of 1.06 and 29.96 Hz) and submitted to an ICA. After removing the ICA components related to eye-movements based on subjective evaluation, the ICA weights from this dataset were applied to dataset with the 0.5–30 Hz band-pass filter. In an additional step, any remaining artifacts were rejected manually. ERPs were calculated for the epochs with a pre-stimulus baseline of 100 ms for each condition separately, i.e., for standards stimuli, intensity deviants and NAD deviants.

Only children with a minimum of 20 deviant trials (25%) per condition remaining were included in the statistical analysis. The resulting mean number of artifact-free trials was identical across deviant conditions (NAD deviants: *M* = 36; SD = 3; intensity deviants: *M* = 36; SD = 4; standards: *M* = 216; SD = 34). Finally, a 10-Hz low-pass filter was applied for data visualization only.

### Statistical analysis

The statistical analyses of the overall effects of the NAD and intensity violations were conducted using the FieldTrip toolbox (Version 20170228; Oostenveld et al., 2011). Additional regression analyses were implemented using the statistical computing software R, Version 4.3.1 (R Core Team, 2022). As a first step, non-parametric, cluster-based permutation tests using dependent-sample *t*-tests were performed for each of the two experimental conditions, comparing ERP responses to the final tones of standard triplets to those in response to NAD deviants and intensity deviants, respectively. The entire epoch (0–800 ms) and all electrodes (except reference and EOG electrodes) were included in the analysis. In order to be included in a spatial cluster, a minimum of two significant neighboring electrodes was set, with spatial neighborhood defined based on the triangulation method. The statistical threshold for a specific time-sample to be included in a cluster was *p* < 0.05. The cluster-statistic permutation test (maxsum approach) was conducted using 1,000 draws from the permutation distribution via Monte-Carlo simulation and an alpha level of *p* < 0.05 (two-tailed).

As a second step, we performed step-wise regression analyses to test whether an identified NAD effect was related to the intensity effect. *Post hoc*, we extracted the mean amplitude values of the NAD and intensity effects for the indicated approximate time window of the dependency effect (195–329 ms) from seven frontal electrodes (F7, F3, Fz, F4, F8, FC3, and FC4) which were identified as part of the clusters in both NAD and intensity conditions. In order to ensure that any such relation was between NAD learning and intensity discrimination specifically and not grounded in interindividual differences with respect to amplitudes of auditory-related ERPs *per se* (which might affect both the NAD and intensity effects), we included the amplitude of the ERP response to standard stimuli as an additional predictor. This amplitude was extracted from the same time window as was used for the NAD and the intensity effect (195–329 ms).

## Results

**FIGURE 2:**
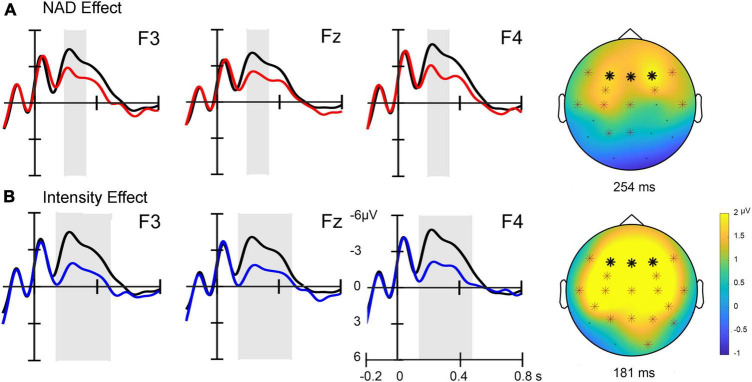
**(A)** Event-related potential (ERP) effects for NAD deviants (red lines) compared to standard stimuli (black lines) at three frontal electrode sites (marked with bold stars in the topographies). A topographic difference map on the right displays the positivity at its maximum with electrode sites included in the cluster marked with stars. **(B)** ERP effects for intensity deviants (blue lines) compared to standard stimuli (black lines) at three frontal electrode sites. A topographic difference map displays the positivity at its maximum with significant electrode sites marked. The sites of the electrodes included in the cluster are marked with stars.

Figure 2 displays the ERP waveforms in response to the final tones of the triplets. The ERPs show a negative peak immediately after the onset of the final tone, which is followed by a short positive peak and a somewhat longer-lasting negativity. Before the baseline period (−100 to 0 ms), another negative peak is visible followed by a short positive peak, which can be related to the presentation of the second tone in the triplet. The short sequence of negative peaks accompanying the rapid presentation of single tones suggests that each tone elicits a characteristic ERP response consisting of obligatory auditory components appropriate for this age group (Ceponiene et al., 2002). Note, however, that these ERP responses to individual tones cannot be disentangled due to our short SOAs of 150 ms (from onset to onset). Importantly, the second tones, for which the responses are visible in the baseline, were similarly distributed from a set of 20 different tones across standard sequences as well as intensity and NAD deviant sequences.

### NAD learning and intensity effects

Averaged ERPs across fronto-central electrode sites show a positive deflection for NAD deviants compared to standard stimuli (cf. Figure 2). The non-parametric cluster-based permutation test revealed this condition difference to be statistically significant (clusterstat *T* = 2.186, *p* < 0.05) within a time window ranging from approximately 195–326 ms relative to the onset of the last tone of the triplet (Figure 2A). For the comparison of standards versus intensity deviants, a similar, somewhat more broadly distributed positivity is visible at fronto-central electrodes. This condition difference is also confirmed statistically by a cluster-based permutation test (clusterstat *T* = 12.933, *p* < 0.01) (cf. Figure 2B) for an approximate time window of 142–479 ms relative to the onset of the last tone in the triplet.

### Regression analysis

**FIGURE 3:**
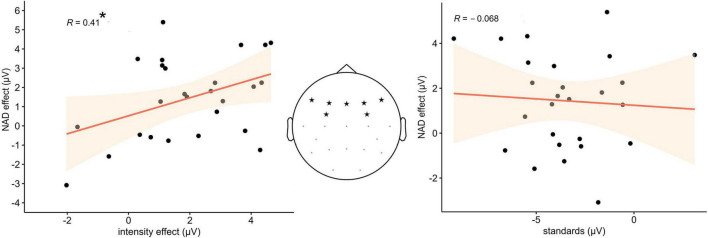
**(Left)** Statistically significant positive relation between the difference amplitude values of the intensity effect and the NAD effect in the ERP data. **(Middle)** Electrode sites from which the amplitude values were extracted. **(Right)** No statistically significant relation between the ERP amplitude of the standard stimuli and the NAD effect.

The step-wise regression revealed that only the intensity effect predicted the NAD effect, while the amplitude in response to the standard stimuli alone did not. Akaike information criterion (AIC) was used to determine whether a factor significantly contributed to the predictive power of the model. Results revealed that of the tested models, the model including the intensity effect as a sole predictor was an at least equal fit to explain the data (AIC 37.62) compared to the full model (AIC 39.44), which included both intensity effect and standard amplitudes, and a significantly better fit compared to the model with only the standard amplitude (AIC 42.2). In the final model, the amplitude of the intensity effect was a significant predictor of the NAD effect amplitude [*F*(1,24) = 4.76, *p* < 0.05], with an explained variance of *R*^2^ = 0.13. Figure 3 displays scatterplots of the results including Pearson correlation coefficients.

## Discussion

The goal of the present study was to evaluate whether NAD learning could be observed in 3-year-old infants for non-linguistic tone sequences, similarly to the processing of linguistic sequences reported previously (Mueller et al., 2019). Additionally, the study probed the relationship between basic auditory processing ability in terms of intensity variation detection and NAD learning. Here, we found that learning of non-adjacent tone sequences is present in 3-year-olds. Yet, in contrast to our original hypothesis derived from findings of linguistic NAD learning at this age, successful learning and deviant detection was evidenced by a positive, not a negative, deflection in the ERP response. Further, the present data suggest that non-linguistic NAD learning is linked to intensity discrimination, similarly to how linguistic NAD learning has been linked to auditory pitch discrimination (Mueller et al., 2012). As a note of caution, given that our sample comprised more male than female participants, we cannot exclude that this bias influenced our findings.

The successful learning of non-linguistic NADs in the present study is consistent with the findings of van der Kant et al. (2020), who reported fNIRS evidence for the learning of NADs in tone sequences, but not in linguistic sequences, in 3-year-olds. Relatedly, two ERP studies reported that ERP indices of linguistic NAD learning decreased in amplitude between the first birthday and the age of 4 years (Mueller et al., 2019; Paul et al., 2021). Given that for the present study, no longitudinal data is available, it remains an open question whether the observed NAD learning effect for non-linguistic auditory stimuli would also decrease with increasing age. Initially, it may seem counterintuitive that sine tones, which are much less relevant and appealing for humans than speech (Vouloumanos and Werker, 2004), yield a more robust learning effect than species-specific communicative sounds. A whole range of experiments with younger infants has indeed suggested the opposite, namely an advantage of speech stimuli (and other communicative signals) over non-speech stimuli for the learning of repetition-based regularities (Ferguson and Lew-Williams, 2016; Rabagliati et al., 2019). We argue here that by the age of 3 years, language and its basic regularities may be so familiar already, and potentially even entrenched, that identifying and learning new language dependencies may require much more effort compared to the acquisition of new dependencies in a non-familiar sound system. Entrenchment, that is, the reduced plasticity of a system after it has settled in a stable state due to extensive exposure, has been brought up as a mechanism that applies to human statistical learning (Bulgarelli and Weiss, 2016; Siegelman et al., 2018) and as a potential explanation for some of the difficulties second language learners experience (MacWhinney, 2016). Thus, NADs in sine tone sequences might be easy to learn for 3-year-olds because they do not interfere with an entrenched rule system that is used for communication. Yet, whether previous experience plays a decisive role in explaining the auditory signal-specific differences modulating the efficiency of NAD learning in our study has to be tackled by further research.

Notably, we expected NAD learning/deviancy detection to be indexed by a negative-going ERP effect as our experiment was closely modeled after Mueller et al. (2019), who reported negative responses for linguistic NAD deviants compared to standards for both 2-year-olds and 4-year-olds. Yet, in our study, both deviant conditions yielded positive-going responses with similar fronto-central distributions. Although the polarity of these responses was unexpected for the current design, they still indexed children’s successful detection of both acoustic and NAD changes. Moreover, positive-going responses for deviancy detection have previously been reported in similar oddball experiments with infants during their first year of life (Friederici et al., 2011; Mueller et al., 2012; Weyers et al., 2022). In addition to younger children often showing positivities and older children negativities in deviancy detection (e.g., Pihko et al., 1999; Cheng et al., 2013, 2015; Reh et al., 2021), several stimulus and experimental factors seem to influence the polarity of the deviancy response. For example, the type and acoustic distance of the tested sound contrasts (e.g., Morr et al., 2002; Cheng et al., 2013, 2015), experimental conditions (e.g., duration of inter-stimulus-interval; Cheour et al., 2002; Kushnerenko et al., 2002), and data-analysis decisions (e.g., Weber et al., 2004; He et al., 2007) have been reported to impact on the polarity of the deviancy response. For the current study, differences in the properties of non-linguistic and linguistic stimuli and the way NADs were encoded are particularly relevant. Here, previous infant studies reported positivities for deviancy detection of smaller stimulus differences and negativities for larger differences (Cheng et al., 2013, 2015). However, quantitative aspects of the difference between correct and incorrect NADs across tone and speech stimuli are difficult to judge. Furthermore, in studies with linguistic stimuli, positive responses have been proposed to reflect acoustic processing and negativities rather phonological processing (Rivera-Gaxiola et al., 2005; Garcia-Sierra et al., 2016; Ferjan Ramírez et al., 2017). Taken together, non-linguistic and linguistic stimuli are likely to trigger different processing levels in listeners’ deviancy detection, resulting in positive responses for both acoustic and NAD changes in pure-tone sequences in the current study.

As hypothesized, we found a predictive relation between the ERP effect reflecting intensity discrimination and the ERP effect reflecting the detection of the NAD deviant. Similar links were reported previously for linguistic NAD learning in 3-month-olds, 4-year-olds, and adult participants, whereby larger responses for pitch discrimination were positively related to the responses for the detection of NAD violations (Mueller et al., 2012, 2019). These studies left unclear, however, whether this relation is specific to the frequency domain, or whether it is indicative of a more general effect linking auditory perception and sequence learning. In our study, the NADs were coded by pitch, while intensity was held constant across all standard stimuli. The feature intensity was thus not relevant for the extraction of the regularity. Nonetheless, participants’ response for intensity discrimination was predictive of their response for NAD violation detection. Interindividual differences in the amplitude of the ERP response to the standard stimuli alone did not contribute to this link. This makes it unlikely that unspecific interindividual differences with respect to the measured ERP amplitudes (Melnik et al., 2017) explain the effects we found. The present results suggest that aspects of auditory processing ability, namely the ability to discriminate sounds based on their intensity, is predictive of the ability to detect NADs in tone sequences. We interpret this as indicative of a general link between auditory perception and stimulus-driven learning. What could it be that links the two? Since intensity discrimination and the detection of tone sequence patterns are not intrinsically related, we suggest that both are linked via a shared process, namely bottom-up auditory attention (Addleman and Jiang, 2019). It has been suggested that stimulus-driven auditory attention is affected by a variety of internal and external factors, which contribute to the salience of a stimulus over space and time. Specifically, pitch and intensity have shown to contribute to this process (Kaya and Elhilali, 2014; Addleman and Jiang, 2019). Following this, our data suggest that there are interindividual differences in how strongly children react to stimulus-driven saliency across different perceptual domains – namely intensity and pitch modulation over time (which could be one way to describe the NADs in the current study in which pitch patterned in complex ways across three items). The underlying link could thus be an attention-based mechanism, modulated by the sensitivity to respond to salient auditory stimuli, be it on the basis of the temporary state the child is in at the particular moment of testing, or a more general individual ability.

## Conclusion

In sum, the current study provided ERP evidence for the ability of 3-year-old children to extract NADs from sine tone sequences. Although the specific ERP pattern (positivity) indicating learning success contrasts with evidence from similar studies from the linguistic domain, the evidence is consistent with the idea that NAD learning in early childhood is present across the linguistic and the non-linguistic domain. Further, we were able to extend the finding of a relation between auditory processing and NAD learning to the domain of non-linguistic tones. We propose that this is due to a shared attentional mechanism linking perception and NAD learning in the present design. Further studies will have to test the pervasiveness of such a potential linking mechanism across modalities and types of input.

## Data availability statement

The raw data supporting the conclusions of this article will be made available by the authors, without undue reservation.

## Ethics statement

The studies involving humans were approved by the Ethics Advisory Board of the Medical Faculty of the University of Leipzig. The studies were conducted in accordance with the local legislation and institutional requirements. Written informed consent for participation in this study was provided by the participants’ legal guardians/next of kin.

## Author contributions

JM: Conceptualization, Data curation, Investigation, Methodology, Project administration, Supervision, Visualization, Writing – original draft. IW: Methodology, Writing – review & editing. AF: Conceptualization, Resources, Writing – review & editing. CM: Conceptualization, Investigation, Methodology, Supervision, Writing – review & editing.
